# TECLA: A temperament and psychological type prediction framework from Twitter data

**DOI:** 10.1371/journal.pone.0212844

**Published:** 2019-03-12

**Authors:** Ana Carolina E. S. Lima, Leandro Nunes de Castro

**Affiliations:** Natural Computing and Machine Learning Laboratory, Mackenzie Presbyterian University, São Paulo, Brazil; The University of Hong Kong, HONG KONG

## Abstract

Temperament and Psychological Types can be defined as innate psychological characteristics associated with how we relate with the world, and often influence our study and career choices. Furthermore, understanding these features help us manage conflicts, develop leadership, improve teaching and many other skills. Assigning temperament and psychological types is usually made by filling specific questionnaires. However, it is possible to identify temperamental characteristics from a linguistic and behavioral analysis of social media data from a user. Thus, machine-learning algorithms can be used to learn from a user’s social media data and infer his/her behavioral type. This paper initially provides a brief historical review of theories on temperament and then brings a survey of research aimed at predicting temperament and psychological types from social media data. It follows with the proposal of a framework to predict temperament and psychological types from a linguistic and behavioral analysis of Twitter data. The proposed framework infers temperament types following the David Keirsey’s model, and psychological types based on the MBTI model. Various data modelling and classifiers are used. The results showed that Random Forests with the LIWC technique can predict with 96.46% of accuracy the Artisan temperament, 92.19% the Guardian temperament, 78.68% the Idealist, and 83.82% the Rational temperament. The MBTI results also showed that Random Forests achieved a better performance with an accuracy of 82.05% for the E/I pair, 88.38% for the S/N pair, 80.57% for the T/F pair, and 78.26% for the J/P pair.

## Introduction

The study of psychological types or temperament lead us to the understanding of how a person relates with the world, either by the choices he makes or the way he absorbs information. For a long time, this theme has been researched and associated with well-being, lifestyle, employment, leadership, study, etc. One way of knowing a person’s psychological type is by submitting him to questionnaires about his habits and choices, for example the MBTI (Myers-Briggs Type Indicator), which returns the psychological type of a person and is based on the studies of Jung and Myers-Briggs, and the Keirsey Temperament Sorter (KTS), which returns a profile associated with the temperament taxonomy created by David Keirsey.

In general, such forms involve many questions and can be biased by the environment in which the respondent is. One way to balance this bias would be to extract information in a passive way, for example, in the interactions (posts, likes, etc.) within social media, a service increasingly present in our daily lives. Social media can be seen as repositories of actions, behaviors and preferences that can be mapped onto psychological features. This occurs due to a user-free content creation, where each person has a role in creating and sharing content [1]. Wiszniewski and Coyne [2] argue that whenever an individual interacts in a social sphere he paints before himself a mask of his identity that becomes even more pronounced as the individual needs to fill in a profile.

The goal of this research is to identify if there are behavioral patterns in the information shared in social media that can be mapped with high precision into the psychological types of the MBTI or the temperaments of Keirsey. This is, therefore, an exploratory paper on the ability of traditional text mining techniques and natural language processing to assist in the extraction and classification of patterns. From our literature review we expand the combinations of text pre-processing techniques and classification algorithms in relation to the papers presented here. We also mapped a database of MBTI results in the Artisan, Guardian, Idealist and Rational types in order to demonstrate the applicability also in the concept of temperament proposed by David Keirsey. In terms of application, it is useful for the preparation of marketing campaigns, more accurate hiring and promotion processes, turnover reduction, improvement of working environment quality, and many other applications related to human capital recruitment, selection and maintenance.

The combination of human behavior research and text/data mining techniques provides insights about the virtual persona, such as his/her influence on others [3, 4], how much they trust one another [5, 6], their life satisfaction [7], personality [8, 1, 9, 10, 11, 12], emotions [13, 14], political preferences [15, 16], emotion and mood state [17, 18], depression [19, 20], disorders [21, 22], among many others.

The goal of automating the prediction of temperament and psychological type is not to replace the use of tests already validated, but, instead, to provide a new tool based on a completely different and passive data to support specialists. More specifically, this research will be based on Twitter data as case study, mainly due to its flexibility in providing open data for collection and analysis. This paper presents a series of classifiers evaluations to map the behavior of social media users, based on their Twitter posts, in relation to the temperament and psychological type and summarize the methodology in a structure called Temperament Classification Framework (TECLA).

To assess the performance of the proposed framework we used a dataset from the literature containing over a million tweets from 1,500 users. Five classification algorithms were evaluated: Naïve Bayes (NB); Support Vector Machines (SVM); Decision Tree (J48); Multilayer Perceptron (MLP); and K-Nearest Neighbors (KNN). We compare these algorithms with Twitter features and three text representation schemes (MRC, LIWC, Apache OpenNLP) to find a suitable combination to determine the temperament and psychological types based on Twitter messages.

The paper is organized as follows. Section 2 provides a brief historical perspective on temperament theories, emphasizing the models proposed by Myers-Briggs and later Keirsey. Section 3 brings a brief review of the works in the literature dealing with the automatic classification of temperament and psychological types. The Temperament Classification Framework (TECLA) is presented in Section 4, and its performance is analyzed in Section 5. The paper is concluded in Section 6 with a general discussion and perspectives for future research.

## A Brief historical perspective on temperament theories

*Temperament* characterizes a set of mental tendencies related to the way someone perceives, analyzes and makes daily decisions [23]. It represents the uniqueness and intensity of psychic affects and the dominant structure of mood and motivation in each individual. It is a form of reaction and sensitivity of a person to the world, which is revealed by his/her attitudes and behaviors, thus composing his/her organic basis [24]. This set of trends is innate, that is, it appears from birth, and is closely linked to biological or physiological determinants, which therefore change relatively little with development [25]. It can change and weakens throughout life, but it is never eliminated [24]. In the present research, *temperament is defined as a set of innate and hereditary tendencies*, *responsible for how one perceives and interacts with the world*.

The literature is filled with different terminologies to refer to temperament, based on the authors’ view of such characteristics. For instance, Hippocrates called it the four humors, Carl Jung, Isabel Myers Briggs and Katharine Cook Briggs called it psychological types, and Carlos Galeno and David Keirsey, called it temperament [25, 26, 27]. We summarize the temperament as a concept that converges to a set of innate characteristics of an individual, closely linked to biological or physiological determinants, which change relatively little during the personal development [25].

We adopted the temperament model proposed by David Keirsey [27] and the psychological types introduced by Myers and Briggs [28]. Keirsey’s model maps temperament into four types: artisan; guardian; idealist; and rational. This model is widely accepted for the understanding of professional trends, thus being potentially applicable in recruitment and selection processes, promising areas for social media data analysis. The Myers and Briggs’ model has a set of 16 psychological types that were investigated and defined from the studies of Carl Jung on the psychological types.

Carl Gustav Jung proposed one of the most comprehensive and well-known temperament typologies in his book *Psychological Types* [29]. Jung analyzed the temperament according to the workings of the mind. For him the mind is composed of an association between *attitudes* and *functions*. The attitudes (*extroversion* (E) and *introversion* (I)) would be the source of psychic energy and the functions correspond to the way each individual acquires and processes information. Jung related four functions, two referring to obtaining information: *sensation* (S) and *intuition* (N); and two for decision-making: *thought* (T) and *feeling* (F) [25]. Then, Isabel Myers and Katheryn Myers Briggs added a new pair of functions: *judgment* (J) and *perception* (P), which assess whether an individual’s orientation to the outside world comes from a *rational* (*judging*) or *irrational* (*perceiving*) function.

D. Keirsey [27] focused his research on the parallel between the Myers-Briggs taxonomy and the observation of temperament in action at the time of choices, behavior patterns, logic and consistency. He assumed that the temperament associated with character forms the personality of the individual; the temperament being innate and the character emergent, developed by the interaction of temperament with the environment. Thus, the types are driven by aspirations and interests, which is what motivates us to live, act, move and play a role in society [27]. He noted that the interests and aspirations are more related to the perception (S-N), totally instinctive, more than to decision-making (T-F), which is fully rational. The sensation (S) can combine with judgment (J) or perception (P), while intuition (N) with feeling (F) or thinking (T). This observation resulted in four temperament types: *Guardian* (SJ); *Artisan* (SP); *Idealist* (NF); and *Rational* (NT) [23, 27].

Although the characteristics of Myers-Briggs model is binary (dichotomic), there are studies that suggest that a better representation would be continuous with degrees of belonging to each function and attitude [30, 31, 32]. The inventory provided by Myers and Briggs aims to determine which of two functions or attitudes is preferred. The score indicates the tendency in the dichotomy. Results with low scores suggest a tension between the opposite pairs rather than an indication of equal preference. However, the tension is unclear whether the equal represents strength in both pair, equal weakness in both areas, or equal neutrality in both areas [33]. We have adopted the binary standard due to our methodology for acquiring a dataset since the disclosure of the MBTI result by a social media user occurs through the label (ENTJ, INFP, etc.), without direct association with the score in each pair.

## Automatic temperament classification: A literature review

Understanding social media users involves the analysis of their behaviors and interactions in social media, like their followers, mentions, messages, friends, photos, videos and comments. Understanding the users means being able to quantify and qualify how they present themselves [34]. The automatic recognition of temperament by means of computational techniques can help many business sectors and social researchers in understanding social media users. To date, there are only a few works related to the automatic temperament/psychological types classification in the literature, that is, Keirsey and MBTI labels. The main reason for the scarcity of works in this area is the difficulty in finding data for training classifiers. This section provides a review of the specific works found in the literature related with these two topics. Although there are many other works addressing the prediction of user characteristics from social media data, these are out of the scope of the present paper.

Luyckx and Daelemans [35] created a 200,000-word Personae corpus consisting of 145 undergraduate student essays about an Artificial Life documentary written in Dutch. Besides, the students submitted their MBTI profile. In this work, the authors performed an authorship attribution and personality prediction. The Memory-Based Shallow Parser (MBSP), n-gram and Lexical features were used to extract the text features. For personality prediction, a 10-fold cross-validation training was performed with a method based on the K-NN algorithm, called TiMBL (Memory-based learning). The experiments contained 84 binary classification tasks, each one for the MBTI dichotomy. The authors concluded that the prediction of introverted-extraverted and intuitive-sensing were fairly accurate, with average F-measures of 65.38% and 61.81%, respectively.

Komisin and Guinn [36] developed a system based on the classification of documents to determine the psychological type according to Myers-Briggs model. In their experiments, they used a Naïve Bayes classifier and Support Vector Machines. Data were collected as part of a postgraduate course in conflict management offered to undergraduate students, in which students performed the MBTI and Best Possible Future Self (BPFS) tests. The BPFS contains self-descriptive elements, in present and future, in different contexts (e.g., work, school, family, finances). Data were collected over three semesters between 2010 and 2011. The n-gram and Linguistic Inquiry and Word Count (LIWC) were used to provide a representation of texts. The authors concluded that the dichotomies Thinking/Feeling (T/F) were predicted with over 75% accuracy for the precision and recall measures using Naïve Bayes with leave-one-out cross validation. For the Intuitive/Sensing (N/S) dichotomy, the LIWC features resulted in less successful predictions. Introversion/Extroversion (I/E) and Judgement/Perception (J/P) did not achieve good precision and recall results.

Brinks and White [37] used various algorithms to detect the Myers-Briggs temperament types in tweets. The aim of the project was to develop a computer system capable of performing the function of the human analyst trained to apply the MBTI based on textual communication. The authors argued that although the results of the MBTI are confidential, many individuals openly reveal their type in a variety of ways and media, including Twitter. They showed that, in a search on Twitter with the term “#INFP” messages were found such as: “I just reread the Myers-Briggs description of my #INFP personality type. It’s scary accurate”. Thus, the data were collected from users that revealed their temperament profiles. 6,358 Twitter users were observed and it were collected two hundred tweets from each. In total, it was analyzed 960,715 tweets. On average, classifiers achieved a precision of 66.25%.

Plank and Hovy [38] collected 1.2 million tweets classified according to the Myers-Briggs system. For these, the authors monitored messages that mentioned any of the 16 types associated with the words Briggs or Myers. Thus, they obtained 1,500 different users, and collected between 100 and 2,000 of their latest tweets, resulting in a corpus of 1.2 million tweets. The authors structured the messages using *n*-grams, in addition to the genre information, tweets count, number of followers, number of followings, among other service features. One goal was to find out which attributes would be more characteristic in each dimension of the Myers-Briggs model. They used logistic regression to analyze the attributes in each dimension and concluded that the data can provide enough linguistic evidence to predict the dimensions reliably: Introversion/Extroversion and Feeling/Thinking.

Verhoeven et al [39] created a MBTI dataset in six languages (Dutch, German, French, Italian, Portuguese and Spanish) with 18.168 users and approximately 34 million tweets in total distributed among the languages. They used the same methodology presented in [38] to collect the data. After the construction of the database, the authors performed classification tests to predict both gender and Myers-Briggs personality dimensions (I/E, N/S, T/F and J/P). For the experiments the authors used 200 tweets per user and discarded those who had fewer than 200 messages. The authors used LinearSVC with standard parameters with *n*-grams. The classification was performed using 10-fold cross-validation. Considering all languages, the average F-measure for the I/E dimension was 67.87%, 73.01% for the N/S dimension, 58.45% for the T/F dimension, and 56.06% for the J/P dimension.

Lukito et al. [40] used Twitter as data source in Indonesia to predict personality and performed an MBTI psychological test with a user base of 97 people. Approximately 240,000 tweets were collected, an average of 2,500 tweets per Twitter user. They selected 15 users for testing and changed the training set size according to the experiment. The classification algorithm used was Naïve Bayes and the messages were structured by *n*-gram and POS-tag. The best result was achieved for the I/E dichotomy with 80% accuracy, the other dichotomies had the same 60% accuracy levels. The authors compared their results with the work proposed by [38], concluding that their proposal was superior for the pairs I/E and J/P, being the latter one of the most difficult to predict.

Lima & de Castro [1] developed a framework called TECLA to predict temperament types (Artisan, Guardian, Idealist, and Rational). The dataset with approximately 29.200 tweets was collected from Twitter. They used LIWC text representation and Twitter user’s account information (like tweets count, number of followers, and number of followings). The authors used NB, KNN, SVM and Decision Tree algorithms to evaluate the proposal. The best accuracy results were in Artisan and Guardian with 87.67% and 83.56%, respectively. The accuracy did not exceed 60.27% for the Idealist temperament and 58.90% for Rational.

Table 1 shows a summary of the papers found, detaching the classification algorithms, main features and performance measures used. It also presents the best results obtained based on the measures adopted. The results of [37, 38, 40], all based on tweets, suggest a higher predisposition for I/E and N/S pairs. The F-measure in [36] was obtained from the Precision and Recall.

**Table 1:** Summary of the papers found related to temperament classification.

	Algorithm	Features	Measure	I/E	N/S	T/F	J/P
[35]	TiMBL	MBSP, n-gram, Lexical features	F-Measure	65.38%	61.81%	49.09%	51.67%
[36]	NB, SVM	n-gram, LIWC	F-measure	I: 9.00% E: 50.00%	N: 75.88% S: 78.42%	F: 75.00% T: 73.00%	J: 84.26% P: 72.93%
[37]	NB, logistic regression and SV classification	n-gram	Accuracy	63.90%	74.60%	60.80%	58.50%
[38]	Logistic regression	n-gram	Accuracy	72.50%	77.40%	61.20%	55.40%
[39]	LinearSVC	n-gram	F-Measure	67.87%	73.01%	58.45%	56.06%
[40]	NB	n-gram, POS-tags	Accuracy	80.00%	60.00%	60.00%	60.00%

## TECLA: The temperament classification framework

The Temperament Classification Framework (TECLA) was developed as an outcome of the use of text mining and natural language processing techniques to classify the temperament or psychological type of social media users. The goal is to provide a modular structure that allows us to use and evaluate different techniques quickly and intuitively. Furthermore, it follows the main steps of KDD (*Knowledge Discovery in Databases*) [41]. Hence, the TECLA has the following modules: *data acquisition module*; *message preprocessing module*; *temperament classification module*; and *evaluation module*. Each one of them will be detailed in the following.

### Data acquisition module

The data acquisition module is responsible for monitoring and receiving information from the users to be classified. For example, in the case of Twitter, it is necessary to obtain usage information, such as number of tweets, number of followers and followed, plus a set of tweets.

### Message pre-processing module

The TECLA framework does not work directly with the tweets, but uses information extracted from them, called meta-attributes. Such information can be divided into two categories: grammatical and behavioral. The behavioral category extracts information about the social media use and is specific to each type of media. In the case of Twitter, it includes the number of tweets, number of followers, followed, favorites, number of listings and number of times the user was favorited. The grammar category considers information from LIWC [42, 43], MRC [44], sTagger [45], or oNLP [46], extracted from the user’s set of messages, similarly to what was proposed in the Polarity Analysis Framework introduced by the authors [14]. Therefore, the message pre-processing module is responsible for extracting meta-attributes from the data (usage and message corpus) and building a new base, called meta-base, from the extracted meta-attributes. The list of meta-attributes used in TECLA is summarized in Table 2.

**Table 2:** Meta-attributes used in the TECLA framework.

Name	Type	Description
A_1_	Behavior	Total number of tweets posted by the user so far
A_2_	Behavior	Number of followers
A_3_	Behavior	Number of followed
A_4_	Behavior	Number of times the user was listed
A_5_	Behavior	Number of times the user was favorited
A_6_	Behavior	Gender
A_7_ to A_94_	Grammatical	If attributes from LIWC
A_7_ to A_19_	Grammatical	If attributes from MRC
A_7_ to A_41_	Grammatical	If attributes from sTagger
A_7_ to A_41_	Grammatical	If attributes from oNLP

### Temperament classification module

**Fig 1:**
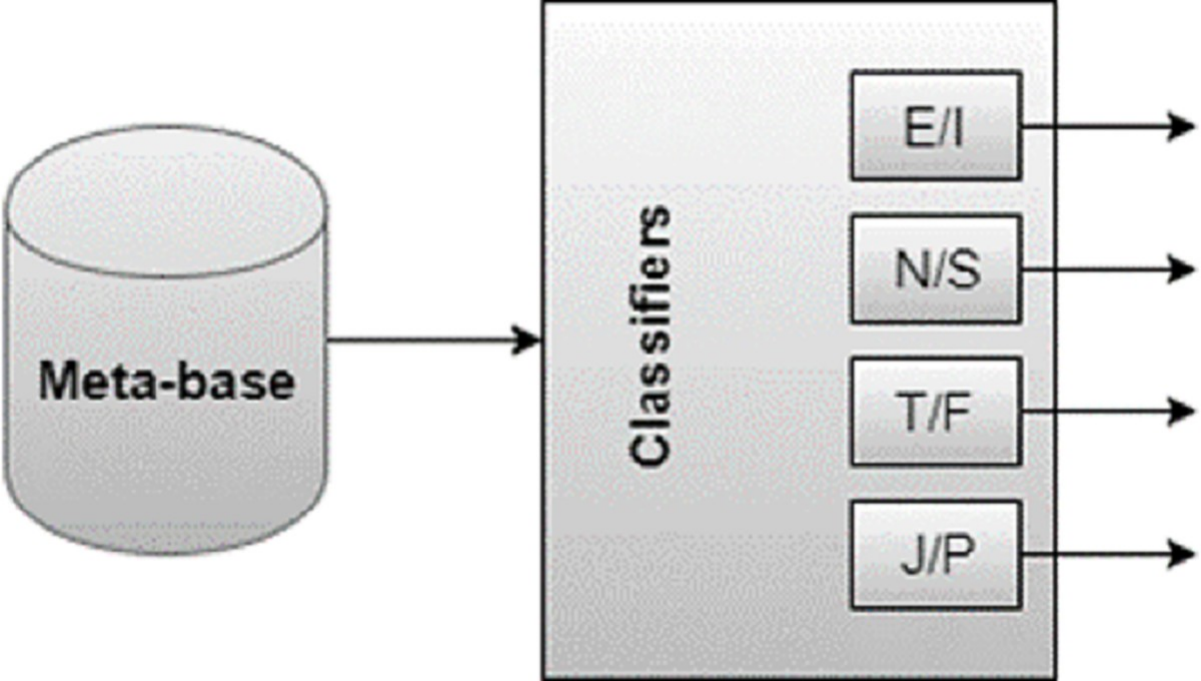
MBTI classification scheme: four decomposing classifiers are trained.

The temperament classification module infers a temperament from the characteristics (meta-attributes) extracted in the previous module. In principle, this module is based on the application of a specific algorithm and can incorporate any kind of classifier. For the classification of the MBTI model, the system was designed with four classifiers (Fig 1) that receive the same data, but is trained to identify the opposing pairs of attitudes and functions. A classifier is trained and responsible for defining the attitude (Extroversion/Introversion—E/I) and the others the functions (Intuition/Sensation—N/S, Thinking/Feeling—T/F, Judgment/Perception—J/P), all trained in isolation. These classifiers were called *decomposing classifiers*.

**Fig 2:**
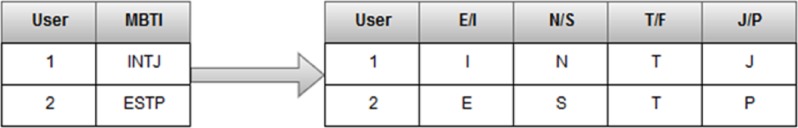
Example of the classifier representation used in TECLA for the MBTI model.

Each of these classifiers is binary, so the answer is either Extroversion or Introversion, Intuition or Sensation, Thought or Feeling, Judgment or Perception. After training, the response of the four classifiers will define the psychological type, e.g., ISTJ or ENFP (Section 2). Therefore, the psychological type of each user was split into four binary classes. The user may be extroverted or introverted, intuitive or sensory, thinker or sentimental, and judgmental or perceptive, as illustrated in Fig 2.

**Fig 3:**
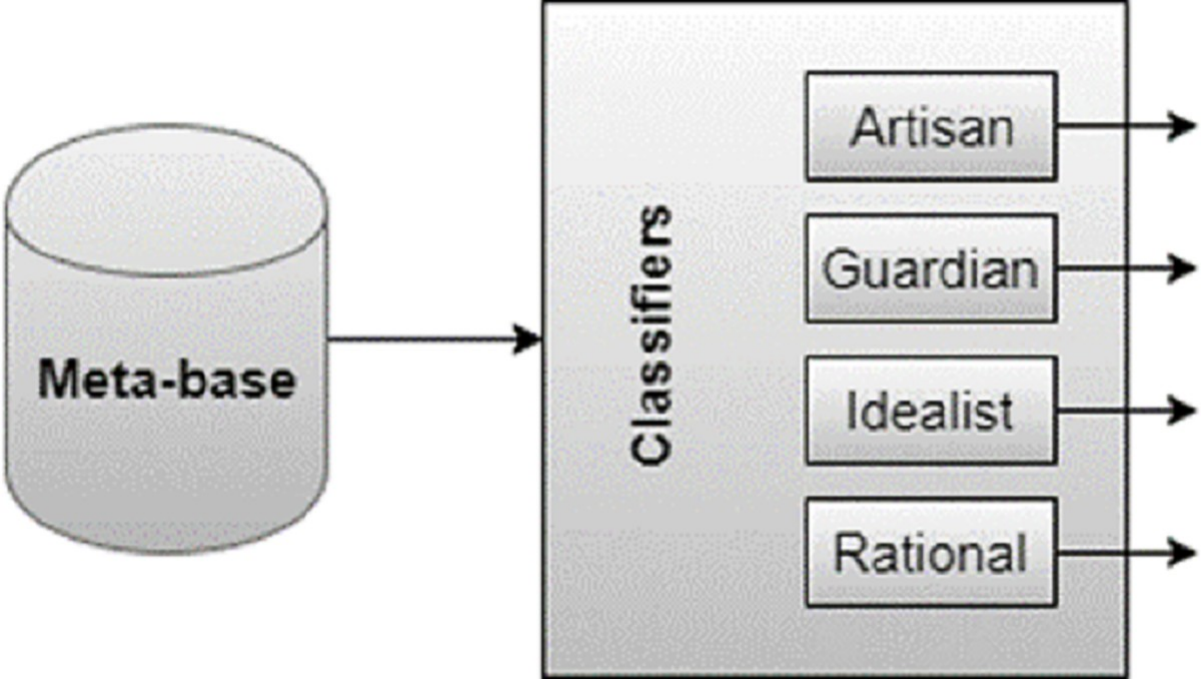
Keirsey classification scheme: four binary classifiers are trained.

For the classification based on the Keirsey model a sequence of classifiers was constructed. As pointed out in [47] one of the strategies to work with multiclass classifiers is the combination of classifiers generated in binary subproblems. With this, there is a decomposition of the problem into binary problems. Separating the problem into binary classifiers can reduce the computational complexity involved in solving the total problem with simpler subtasks. In this case, the classifier has the same scheme shown in Fig 1, however, the first classifier that returns the result “1” will determine the class of the object, as illustrated in Fig 3.

### Evaluation module

In order to measure the TECLA performance, it was used the accuracy, F-measure, which involves precision and recall, and the area under the ROC curve (AUC). Accuracy is the number of objects correctly classified over the sum of all objects. The F-measure represents the harmonic mean between precision and recall, where precision is the percentage of a class correctly classified and recall is the number of objects correctly classified over the total number of objects that really belong to that class [48, 49]

## Performance assessment

The goal of this study is to design a temperament predictor that can infer the temperament of a certain individual (social media user) based on what he writes in the social media, instead of applying him a specific temperament test. This is a very interesting and promising approach, because it allows one to know someone’s temperament in spontaneous situations. To assess the performance of TECLA we used a recent, public dataset with over one million tweets.

### Data acquisition

The database used comes from the [38] paper, in which the Twitter users are classified according to the psychological types of Myers-Briggs. The dataset contains 1.2 million tweets from 1,500 users. The number of tweets varies from one user to another. To be part of the database a user needs to have at least 100 tweets and we downloaded at most 2,000 tweets per user. The attributes available and useful are: MBTI; gender; number of followers; number of tweets; number of favorites; and number of listings. Table 3 shows the user distribution for each psychological type of the Myers-Briggs taxonomy. Although considered rare, the intuitive types, especially the INFP and INTJ, were the most common types within the collected database. By contrast, the sensory types (ESFJ, ESTJ, ESFP, ESTP, ISFP, ISTP, ISFJ and ISTJ) accounted for only about 21% of the data.

**Table 3:** Distribution of users for each MBTI type.

I	ISTJ	75	ISFJ	77	INFJ	257	INTJ	193
	ISTP	22	ISFP	51	INFP	175	INTP	111
E	ESTP	15	ESFP	26	ENFP	148	ENTP	70
	ESTJ	36	ESFJ	36	ENFJ	106	ENTJ	102

The ratio between each element of the E/I, N/S, T/F, and J/P pairs can be seen in Table 4. There is a clear imbalance between the N/S pair, which may reflect the classification results. However, for this study, no class balancing was performed because it would imply a reduction in the number of users in other pairs.

**Table 4:** Ratio between the various MBTI types of users.

E/I	539 (35.93%)	961 (64.07%)
N/S	1162 (77.47%)	338 (22.53%)
T/F	624 (41.60%)	876 (58.40%)
J/P	882 (58.80%)	618 (41.20%)
Female/Male	939 (62.60%)	561 (37.40%)

To evaluate the Keirsey model, each MBTI type was mapped into its model (Artisan, Guardian, Idealist and Rational). Table 5 describes the number of users by temperament. The Artisan and Guardian classes have the smallest number of users, because of the predominance of intuitive in the database (Idealists and Rational).

**Table 5:** Proportion of users by temperament in the dataset collected.

Temperament	Count	Percentage
Guardian (ISTP, ISFP, ESTP, ESFP)	224	14,93%
Artisan (ISTJ, ISFJ, ESTJ, ESFJ)	114	7,60%
Idealist (INFJ, INFP, ENFJ, ENFP)	686	45,73%
Rational (INTJ, INTP, ENTJ, ENTP)	476	31,73%

### Pre-processing

The attributes provided by the Plank dataset are called behavior attributes, in reference to the behavior of users in the microblog. Table 6 shows the average value of the behavior attributes for each temperament (followers, statuses, favorites, listed and gender). In all temperaments/psychological types the predominant gender was female. In the N/S pair we emphasize the fact that the sensorial ones have, on average, more followers and tweet more frequently, although this is the function with fewer representatives in the database (only 22.53%). The difference between Guardians and Artisans, both sensory, is greater in relation to the number of followers and listed count. On the other hand, among the intuitive there is a greater balance in the way of using the microblog.

**Table 6:** Average (mode) value for each attribute extracted by Plank.

Myers-Briggs	Total	Avg. Followers	Avg. Statuses	Avg. Favorites	Avg. Listed	Gender (F/M)
Extroversion	539	1549.59	14587.85	4185.09	44.32	325/214
Introversion	961	1694.10	17279.32	4928.68	30.66	614/347
Sensing	338	2851.14	18976.33	5312.95	51.95	229/109
Intuition	1162	1290.51	15537.25	4471.99	30.80	710/452
Thinking	624	1529.57	15959.86	4157.91	25.99	340/284
Feeling	876	1722.38	16563.16	5020.20	42.39	599/277
Judging	882	2034.39	15359.27	4150.58	40.52	564/318
Perceiving	618	1082.41	17672.17	5390.64	28.50	375/243
Keirsey	Total	Avg. Followers	Avg. Statuses	Avg. Favorites	Avg. Listed	Gender (F/M)
Guardian	224	3924.24	18501.26	4864.42	68.96	153/71
Artisan	114	742.60	19909.79	6194.29	18.53	76/38
Idealist	686	1201.93	15959.14	4720.33	33.49	459/227
Rational	476	1418.18	14929.22	4114.08	26.93	251/225

### Experimental results

All tests were performed with 10 runs of a *k*-fold cross-validation (*k* = 5). First the results will be presented for the Keirsey model, then the MBTI model. In both cases, it is expected to show the ability of the classifiers to infer each of the classes, that is, if from the input data it is possible to identify an Artisan, Guardian, Idealist or Rational person, or, based on the MBTI model the pairs E/I, N/S, T/F, J/P. In all cases the measures adopted to evaluate the classifiers were the *accuracy per class* (*percentage of correct classification per class*, *ACC*), the *F-measure* (*F*), which is the harmonic mean between *Precision* and *Recall*, as discussed previously, and the *area under the curve* (AUC). The AUC is a summary of the ROC curve (sensitivity versus specificity), and high levels in AUC indicate that, on average, the true positive rate is higher than the false positive rate. The following classifiers were evaluated: AdaBoost; Bagging; J48; Naïve Bayes; Random Forest; and SVM. All classifiers used are from the Python library Scikit-learn 0.19.1 with default settings. We used a workstation with an Intel Core i5-3210M @ 3,10 GHz, 3 MB smart cache, quad-core on hyper-threading, 6 GB RAM memory, 904 GB HD @ 5400 RPM and Windows 8.1 operation system.

#### Results for the keirsey model

The following tables show the test results for the Keirsey model: Artisan; Guardian; Idealist; and Rational. The goal is to answer the following question: “*Is it possible to infer the user’s temperament based on his posts*?”. Our tests began with an attribute analysis to understand the best possible configuration. We performed a ranking of the importance of the attributes based on the information gain to perform attribute selection tests and analyze the best results by observing the accuracy and F-measure. Note that our technique separates binary classifiers for each temperament, so the results are divided into ACC, F-measure for class 0 ("No", which means does not have the temperament), F-measure for class 1 ("Yes", which means has the temperament), and AUC of positive result (“Yes”).

Our first analysis refers only to the Twitter attributes and Table 7 below summarizes these results. It is possible to note that, in general, there is a tendency for the classifiers to choose the "No" class, which is the predominant class. Thus, the F-measure for the "Yes" is low. By comparing the ACC and AUC the best result was achieved with the Random Forest using the 5 attributes (total number of tweets posted by the user so far, number of followers, number of followed, number of times the user was listed, and number of times the user was favorited).

**Table 7:** Accuracy (ACC), F-measure (F) and AUC for Twitter with 5 features.

Algorithm	ACC	F-measure (No)	F-measure (Yes)	AUC
AdaBoost	75.46%±0.38%	83.86%±0.34%	9.94%±9.94%	58.32±0.64
Bagging	81.69%±0.31%	86.85%±0.34%	40.10%±40.10%	77.14±0.52
J48	76.66%±0.38%	84.62%±0.50%	22.35%±22.35%	54.75±0.57
NaiveBayes	48.67%±2.06%	47.78%±2.04%	31.67%±31.67%	51.89±0.16
RandomForest	86.64%±0.26%	90.05%±0.26%	68.51%±68.51%	83.81±0.91
SVM	74.88%±0.32%	81.07%±1.31%	12.88%±12.88%	49.98±0.02

We proceeded testing in scenarios in which the Twitter attributes would not be available, but only the text of tweets. For this case, we have tested three text structuring techniques separately, as mentioned in the pre-processing section: MRC, LIWC and oNLP. Note that the performance had the same behavior of the previous evaluation with a low F-measure for the "Yes" class, indicating the trend in the classifiers for one of the classes. Table 8 presents the best performance with 9 attributes with the Random Forest (87.48%±0.25%) and Bagging (83.23%±0.42%) algorithms. The combination SVM + MRC Features was not successful, because the algorithm could not identify patterns for the class Yes (0.00±0.00%).

**Table 8:** Accuracy (ACC), F-measure (F) and AUC for MRC with 9 features.

Algorithm	ACC	F-measure (No)	F-measure (Yes)	AUC
AdaBoost	75.41%±0.50%	83.67%±0.58%	10.33%±2.51%	61.72±1.04
Bagging	83.02%±0.35%	87.78%±0.37%	44.79%±1.27%	80.58±0.2
J48	77.34%±0.83%	84.95%±0.59%	28.69%±2.67%	59.70±0.79
NaiveBayes	72.69%±0.11%	77.29%±0.34%	22.94%±0.63%	57.52±0.24
RandomForest	87.48%±0.25%	90.74%±0.28%	69.81%±0.86%	86.26±0.35
SVM	75.00%±0.00%	84.87%±0.00%	0.00±0.00%	50.00±0.00

The LIWC showed a better performance, which could be noticed in the AUC measure. By analyzing the results we observed that the Random Forest performance is usually superior; the best accuracy was 87.99%±0.29% with 25 attributes (Table 9). In general, there was no significant change in accuracy and the choice for these attributes was due to the F-measure (Yes). However, there was substantial improvement in the AUC value. Thus, the best performance was obtained by the Random Forest with 25 attributes: 91.14%±0.13% for the F-measure (No); and 70.52%±0.81% for the F-measure (Yes).

**Table 9:** Accuracy (ACC) and F-measure (F) for LIWC with 25 features.

Algorithm	ACC	F-measure (No)	F-measure (Yes)	AUC
AdaBoost	75.75±0.27	83.46%±0.87%	17.23%±1.33%	86.83±2.59
Bagging	84.38±0.18	88.78%±0.26%	49.76%±1.65%	84.06±1.53
J48	83.71±0.47	87.75%±0.67%	61.21%±1.08%	87.82±0.82
NaiveBayes	67.49±0.13	75.87%±0.09%	35.90%±0.48%	87.54±0.85
RandomForest	87.91±0.13	91.14%±0.13%	70.52%±0.81%	86.83±2.59
SVM	76.21±0.38	83.95%±0.35%	11.72%±0.46%	84.06±1.53

Similarly to the Twitter and MRC results for oNLP, Bagging and Random Forest (also J48) achieved an AUC above 70%, indicating a better identification of “Yes”. By observing the other measures, again the Random Forest algorithm had the best performance with the oNLP 24 attributes (Table 10). Therefore, the average accuracy was 87.60%±0.33%, the average F-measure (No) was 90.95%±0.31%, the average F-measure (Yes) was 69.68%±0.63% and AUC was 86.12%±0.76%.

**Table 10:** Accuracy (ACC), F-measure (F) and AUC for ONLP with 24 features.

Algorithm	ACC	F-measure (No)	F-measure (Yes)	AUC
AdaBoost	75.36%±0.46%	82.34%±0.94%	12.69%±2.84%	59.48±0.11
Bagging	83.54%±0.46%	88.30%±0.47%	44.23%±1.06%	80.71±0.63
J48	83.26%±0.18%	87.40%±0.29%	61.09%±1.75%	76.28±0.72
NaiveBayes	71.31%±0.07%	78.18%±0.14%	27.52%±0.74%	59.35±0.27
RandomForest	87.60%±0.33%	90.95%±0.31%	69.68%±0.63%	86.12±0.76
SVM	75.60%±0.30%	83.87%±0.27%	9.73%±0.68%	51.49±0.41

Based on its superior performance for all text representation mechanisms, Table 11 details the results of the Random Forest algorithm. By observing the different text representations, the best classification result occurred in the Artisan temperament with 96.46%±0.27% of accuracy for LIWC (25 attributes). These results suggest that the system can be more precise to find what is not Artisan, with all features with an average F-measure of 97.60%±0.24% for Twitter, 98.09%±0.22% for MRC, 98.11%±0.14% for LIWC and 98.08%±0.13% for oNLP. This can also be observed for the Guardian with F-measure (No) of 94.66%±0.30% for Twitter, 95.42%±0.24% for MRC, 95.61%±0.24% for LIWC and 95.51%±0.25% for oNLP. For the idealist the classifier was able to better discriminate the two classes. In the best scenario (LIWC 25 features) the F-measure (No) was 81.47%±0.50% and F-measure (Yes) 74.89%±0.87%. The AUC measure remained constant in all temperament types, around 80%, indicating a low false positive rate.

**Table 11:** Accuracy (ACC), F-measure (F) and AUC for the Random Forest.

Twitter 5 attributes				
	Artisan	Guardian	Idealist	Rational
ACC	95.53%±0.44%	90.63%±0.53%	77.27%±0.59%	80.27%±0.70%
F-measure (No)	97.60%±0.24%	94.66%±0.30%	79.60%±0.49%	86.15%±0.50%
F-measure (Yes)	67.32%±3.05%	61.71%±2.39%	74.32%±0.81%	65.72%±1.45%
AUC	84.08±2.55	82.45±1.13	85.07±0.53	83.65±0.72
MRC 9 attributes				
	Artisan	Guardian	Idealist	Rational
ACC	92.92%±0.30%	87.33%±0.46%	73.09%±0.91%	77.73%±1.18%
F-measure (No)	98.09%±0.22%	95.42%±0.24%	80.07%±0.74%	88.00%±0.62%
F-measure (Yes)	72.54%±3.64%	63.60%±2.42%	74.00%±1.19%	67.05%±2.20%
AUC	89.97±1.63	83.80±1.27	86.33±0.85	84.93±0.95
LIWC 25 attributes				
	Artisan	Guardian	Idealist	Rational
ACC	96.46%±0.27%	92.19%±0.44%	78.68%±0.61%	83.82%±0.70%
F-measure (No)	98.11%±0.14%	95.61%±0.24%	81.47%±0.50%	89.04%±0.45%
F-measure (Yes)	72.54%±2.85%	64.54%±2.75%	74.89%±0.87%	69.13%±1.58%
AUC	86.83±2.59	84.06±1.53	87.82±0.82	87.54±0.85
oNLP 24 attributes				
	Artisan	Guardian	Idealist	Rational
ACC	96.40%±0.20%	92.01%±0.50%	78.29%±1.00%	82.83%±0.90%
F-measure (No)	98.08%±0.13%	95.51%±0.25%	81.09%±1.01%	88.42%±0.61%
F-measure (Yes)	71.46%±2.29%	63.73%±2.57%	74.50%±1.53%	66.75%±1.82%
AUC	86.94±0.52	87.16±1.02	87.03±0.84	86.94±1.17

#### Results for the MBTI Model

The second set of results presented here are for the decomposed classifiers for the MBTI model. Each classifier is responsible for one of the MBTI pairs. In all cases, the same classification algorithm will be run for all classifiers. The goal is to answer the following question: *Is it possible to identify the user's psychological attitudes and functions based on what he/she writes in social media*? If it is possible, then a deeper understanding of the virtual persona can be achieved by analyzing social media data. As our previous analysis with the Keirsey model prediction, we also performed an attribute analysis for the MBTI model prediction. Table 12 summarizes the results of the Twitter attributes’ evaluation. Both F-measure (No) and F-measure (Yes) have a value less than 70%, except for Bagging (71.97%±0.34%) and Random Forest (79.29%±0.23%) with 5 attributes, that is, with all the original attributes of the dataset. Both algorithms also achieved high AUC values indicating a good performance for the positive class.

**Table 12:** Accuracy (ACC), F-measure (F) and AUC for Twitter with 5 features in the MBTI prediction.

Algorithm	ACC	F-measure (No)	F-measure (Yes)	AUC
AdaBoost	65.46%±0.24%	38.81%±2.94%	56.00%±1.61%	58.14±0.49
Bagging	75.12%±0.17%	65.97%±1.03%	71.97%±0.34%	77.45±0.36
J48	66.66%±0.26%	47.52%±1.85%	60.34%±2.44%	58.53±0.45
NaiveBayes	59.98%±0.24%	52.06%±0.39%	31.39%±1.52%	51.11±0.07
RandomForest	81.54%±0.09%	78.71%±0.80%	79.29%±0.23%	84.81±0.20
SVM	65.73%±0.00%	37.01%±0.00%	53.29%±0.00%	49.99±0.03

For the MRC attributes (Table 13), we observed a better performance with 16 attributes. As in the Twitter case attributes, the Bagging and Random Forest algorithms also had a better performance, mainly when we compare the AUC, 81.26±0.46 for Bagging and 87.06±0.25 for the Random Forest. Also, the F-measure, 70.02%±1.15% / 74.46%±0.49% for Bagging and 78.80%±0.62% / 79.13%±0.37% for the Random Forrest. In general, as in the Twitter attributes, the performance of the classifiers was higher when compared with the Keirsey model classification in relation to the F-measure balance.

**Table 13:** Accuracy (ACC), F-measure (F) and AUC for MRC with 16 features in MBTI prediction.

Algorithm	ACC	F-measure (No)	F-measure (Yes)	AUC
AdaBoost	65.75%±0.25%	45.43%±1.07%	53.56%±1.60%	58.10±0.16
Bagging	77.51%±0.13%	70.02%±1.15%	74.46%±0.49%	81.26±0.46
J48	69.93%±0.61%	59.03%±2.11%	65.66%±2.98%	62.76±0.81
NaiveBayes	63.47%±0.11%	49.62%±0.12%	54.39%±0.29%	56.18±0.17
RandomForest	81.83%±0.09%	78.80%±0.62%	79.13%±0.37%	87.06±0.25
SVM	64.72%±0.00%	38.15%±0.00%	40.20%±0.00%	50.00±0.00

The results for the LIWC attributes analysis, presented in Table 14, show a better performance (AUC) for 24–28 attributes with the best result for 27 attributes associated with the Bagging (82.85±0.43), J48 (77.04±0.43) and Random Forest (87.79±0.56) algorithms. This suggests that the LIWC attributes may better characterize the problem when compared with the previous results also for the Naïve Bayes, AdaBoost and SVM.

**Table 14:** Accuracy (ACC), F-measure (F) and AUC for LIWC with 27 features in MBTI prediction.

Algorithm	ACC	F-measure (No)	F-measure (Yes)	AUC
AdaBoost	65.73%±0.28%	47.94%±0.52%	56.47%±2.03%	59.70±0.33
Bagging	78.45%±0.22%	70.97%±0.77%	75.39%±0.70%	82.85±0.43
J48	77.71%±0.52%	72.33%±2.00%	76.73%±0.66%	77.04±0.43
NaiveBayes	61.32%±0.08%	51.82%±0.11%	54.65%±0.37%	58.80±0.15
RandomForest	82.58%±0.08%	79.61%±0.61%	79.92%±0.39%	87.79±0.56
SVM	64.83%±0.04%	38.53%±0.43%	41.42%±0.53%	50.06±0.12

As with LIWC, the oNLP (Table 15) results were satisfactory for Bagging, J48 and Random Forest, mainly for 22 attributes. The highest accuracy was 82.15%±0.14% for the Random Forrest. The Naïve Bayes classifier had the worst accuracy level with only 60.69%±0.13%. AdaBoost and SVM achieved, respectively, 65.02%±0.16% and 64.75%±0.15% of accuracy. Comparing the AUC results, the SVM had the worst performance.

**Table 15:** Accuracy (ACC), F-measure (F) and AUC for ONLP with 22 features in MBTI prediction.

Algorithm	ACC	F-measure (No)	F-measure (Yes)	AUC
AdaBoost	65.02%±0.16%	37.99%±1.41%	58.11%±1.51%	57.08±0.46
Bagging	77.73%±0.22%	69.60%±0.70%	74.74%±0.58%	80.95±0.40
J48	78.09%±0.36%	73.93%±0.80%	76.85%±0.10%	76.70±0.19
NaiveBayes	60.69%±0.13%	51.02%±0.28%	58.09%±0.40%	55.11±0.42
RandomForest	82.15%±0.14%	79.08%±0.82%	79.56%±0.27%	87.02±0.28
SVM	64.75%±0.15%	38.15%±0.21%	43.72%±0.60%	49.95±0.13

Table 16 details the Random Forest algorithm results due to its overall superior performance. For the studied database it was possible to predict the E/I pair with a mean average accuracy of 82.05%±0.65% for the oNLP features. The F-measure (No) indicates the first letter in the pair. In the E/I case, the Random Forest with oNLP features had an F-measure for Extroversion of 87.12%±0.44% and 70.38%±1.26% for Introversion. The pair S/N achieved 88.38%±0.68% of accuracy also with oNLP. The F-measure for N (intuition) was 92.66%±0.41% and 72.13%±1.94% for S (Sensation). In T/F the accuracy was 80.57%±0.80% for LIWC with 27 attributes, 84.49%±0.63% of F-measure to F (Feeling) and 74.01%±1.15% of T (Thinking) F-measure. The pair J/P had the lowest accuracy of 78.26%±0.79% (LIWC with 27 attributes). The precision was better in J (Judging) with 81.49%±0.66% of F-measure. Like the Keirsey type prediction, the AUC indicates a good performance of true positive in relation to false positive rate.

**Table 16:** Accuracy (ACC), F-measure (F) and AUC for the Random Forest in MBTI prediction.

Twitter 5 attributes				
	E/I	S/N	T/F	J/P
ACC	80.82%±0.74%	87.65%±0.96%	79.77%±0.79%	77.93%±0.93%
F-measure (No)	85.85%±0.56%	71.91%±2.58%	83.52%±0.66%	73.54%±1.28%
F-measure (Yes)	70.23%±1.10%	92.09%±0.59%	73.80%±1.14%	81.06%±0.75%
AUC	85.22±0.81	85.33±0.85	85.09±0.78	83.62±1.21
MRC 16 attributes				
	E/I	S/N	T/F	J/P
ACC	81.39%±0.74%	87.32%±0.66%	78.74%±0.66%	77.89%±1.50%
F-measure (No)	86.59%±0.56%	70.77%±1.84%	82.95%±0.52%	72.98%±1.68%
F-measure (Yes)	69.56%±1.14%	91.90%±0.40%	71.78%±0.98%	81.29%±1.36%
AUC	87.85±0.59	86.96±0.93	86.79±0.38	86.64±0.44
LIWC 27 attributes				
	E/I	S/N	T/F	J/P
ACC	81.89%±0.66%	88.17%±1.00%	80.57%±0.80%	78.26%±0.79%
F-measure (No)	87.03%±0.49%	71.54%±2.43%	84.49%±0.63%	73.66%±1.06%
F-measure (Yes)	70.04%±1.05%	92.54%±0.63%	74.01%±1.15%	81.49%±0.66%
AUC	87.86±0.43	87.35±1.69	87.94±0.74	88.02±0.65
oNLP 22 attributes				
	E/I	S/N	T/F	J/P
ACC	82.05%±0.65%	88.38%±0.68%	80.01%±0.89%	77.89%±1.03%
F-measure (No)	87.12%±0.44%	72.13%±1.94%	84.07%±0.65%	73.22%±1.16%
F-measure (Yes)	70.38%±1.26%	92.66%±0.41%	73.15%±1.44%	81.17%±0.94%
AUC	86.94±0.52	87.16±1.02	87.03±0.84	86.94±1.17

### Comparing with results from the literature

Finally, in Table 17 we compare our Keirsey and MBTI results with the literature. By analyzing the results from [38] our performance was superior for all MBTI pairs. We have also been more effective in the I/E and N/S pairs, however the use of Random Forest combined with other forms of text representation has promoted better performance for T/F and J/P pairs. For the classification results of Keirsey temperaments we compared with previous results obtained in the first steps to build this tool. In this case, we have also achieved an increase in performance. The F-measure in [36] was obtained from the Precision and Recall.

**Table 17:** Comparing with MBTI and Keirsey Results from the Literature.

	Algorithm	Features	Measure	I/E	N/S	T/F	J/P
[35]	TiMBL	MBSP, n-gram, Lexical features	F-Measure	65.38%	61.81%	49.09%	51.67%
[36]	NB, SVM	n-gram, LIWC	F-measure	I: 9.00% E: 50.00%	N: 75.88% S: 78.42%	F: 75.00% T: 73.00%	J: 84.26% P: 72.93%
[37]	NB, logistic regression and SV classification	n-gram	Accuracy	63.90%	74.60%	60.80%	58.50%
[38]	Logistic regression	n-gram	Accuracy	72.50%	77.40%	61.20%	55.40%
[39]	LinearSVC	n-gram	F-Measure	67.87%	73.01%	58.45%	56.06%
[40]	NB	n-gram, POS-tags	Accuracy	80.00%	60.00%	60.00%	60.00%
TECLA (MBTI)	Random Forest	LIWC, oNLP	Accuracy	82.05%	88.38%	80.57%	78.26%
TECLA (MBTI)	Random Forest	LIWC, oNLP	F-measure	I: 87.2% E: 70.38%	N: 92.66% S: 72.13%	F: 84.49% T: 74.01%	J: 81.49% P: 73.66%
	Algorithm	Features	Measure	Artisan	Guardian	Idealist	Rational
Lima and de Castro (2016)	SVM, KNN(2)	LIWC	Accuracy	87.67%	83.56%	60.27%	58.90%
TECLA (Keirsey)	Random Forest	LIWC	Accuracy	96.46%	92.19%	78.68%	83.82%

## Discussion and future trends

The purpose of this paper was threefold: to provide a brief historical review on temperament theories; to make a brief survey of machine-learning research on temperament and psychological type prediction; and to investigate the temperament and psychological types prediction based on data produced by social media users. In this latter contribution, the hypothesis this work tries to validate is if it is possible to predict the virtual persona temperament without using a questionnaire, that is, to use artificial intelligence techniques to understand and classify the profile of users based on what they share and how they behave in social media. The importance of this tool lies in trying to lessen a possible bias provided by questionnaires, when a user knows he is being explicitly evaluated.

From the literature review we seek to extend our previous results [14], both on text processing techniques and algorithms for building predictive models. With this, we present a set of results based on the combination of different text structuring techniques and classification algorithms. Derived from the proposals presented by [37] and [38], on Twitter data, we aim to identify the ability of the models to estimate the temperament typology proposed in [27].

User analysis was performed using the database provided in [38], composed of MBTI results and transformed into the Keirsey model, thus performing classification tests for both models. The results pointed to the use of Random Forests with LIWC structuring for the Keirsey model (96.46% of accuracy for Artisan, 92.19% of accuracy for Guardian, 78.68% of accuracy for Idealist, 83.82% of accuracy for Rational), and LIWC or oNLP for the MBTI (82.05% accuracy for E/I pair, 88.38% accuracy for S/N pair, 80.57% accuracy for T/F pair and 78.26% accuracy for J/P pair).

We believe in the importance of understanding the behavior of users on social media, and we also believe that information such as psychological types can help in this regard. This information can serve as input to many profiling systems in various areas. Here, we did an exploratory study aimed at understanding the potential of machine learning techniques for temperament identification. We would like to expand this research to new databases both from Twitter and other social media in order to explore the framework potential. We would also like to present case studies applying TECLA to different groups of users, and thus answer questions such as: What are the profiles of people who talk about the same subject? What is the profile of people who watch a TV show, movie or series? What is the profile of people who consume a specific product or service?

Finally, further research will also assess the computational scalability of TECLA when using High Performance Computing (HPC) platforms. We performed some preliminary experiments in this direction with one of the best scenarios obtained in this paper (i.e., Random Forest for the Keirsey model and Twitter 5 features) using an Intel Xeon Platinum 8160 processor @ 2.10 GHz, each one with 24 physical cores (48 logical) and 33 MB of cache memory, 190 GB of RAM and obtained a significant gain in performance. As social media data arrives continually, a comprehensive set of experiments will be performed to assess the scalability of TECLA in HPC platforms.
